# Adverse events during intrahospital transport of critically ill patients in a large hospital

**DOI:** 10.5935/0103-507X.20190003

**Published:** 2019

**Authors:** Viviane Cordeiro Veiga, Natalia Fioravanti Postalli, Thais Kawagoe Alvarisa, Phillipe Pereira Travassos, Raquel Telles da Silva Vale, Cleyton Zanardo de Oliveira, Salomón Soriano Ordinola Rojas

**Affiliations:** 1 Grupo de Transporte, Hospital BP - A Beneficência Portuguesa de São Paulo - São Paulo (SP), Brasil.; 2 Unidade de Terapia Intensiva Neurológica, Hospital BP - A Beneficência Portuguesa de São Paulo - São Paulo (SP), Brasil.; 3 Bioestatística, Centro de Ensino e Pesquisa, Hospital BP - A Beneficência Portuguesa de São Paulo - São Paulo (SP), Brasil.

**Keywords:** Patient transfer, Quality, Risk factors, Hypnotics and sedatives, Vasodilator agents

## Abstract

**Objective:**

To describe the incidence of clinical and non-clinical events during intrahospital transport of critically ill patients and to analyze the associated risk factors.

**Methods:**

Cohort study with retrospective data collected from October 2016 to October 2017. All cases of intrahospital transport for diagnostic and therapeutic purposes in a large hospital with six adult intensive care units were analyzed, and the adverse events and related risk factors were evaluated.

**Results:**

During the study period, 1,559 intrahospital transports were performed with 1,348 patients, with a mean age of 66 ± 17 years and a mean transport time of 43 ± 34 minutes. During transport, 19.8% of the patients were using vasoactive drugs; 13.7% were under sedation; and 10.6% were under mechanical ventilation. Clinical events occurred in 117 transports (7.5%), and non-clinical events occurred in 125 (8.0%) transports. Communication failures were prevalent; however, the multivariate analysis showed that the use of sedatives, noradrenaline and nitroprusside and a transport time greater than 36.5 minutes were associated with adverse clinical events. The use of dobutamine and a transport time greater than 36.5 minutes were associated with non-clinical events. At the end of transport, 98.1% of the patients presented unchanged clinical conditions compared with baseline.

**Conclusion:**

Intrahospital transport is related to a high incidence of adverse events, and transport time and the use of sedatives and vasoactive drugs were related to these events.

## INTRODUCTION

Studies on intrahospital transport (IHT) have been conducted since the 1970s, and since then, the numbers of descriptive analyses and analyses of risks during transport have been steadily increasing. Management of critically ill patients in the intensive care unit (ICU) requires investigations and therapeutic procedures leading to numerous transports outside the ICU.^(^^1,2^^)^ Studies show that adverse events occur in 6% to more than 70% of IHTs performed. When limiting the definition of adverse events to changes in vital signs, unplanned extubations or cardiorespiratory arrests, this rate approaches 8%.^(^^3^^)^

Several analysis methods have contributed to the identification of risks related to IHT, such as those in epidemiological studies and analyses by intensive care societies. In these studies, the adverse events associated with transport were correlated with patient, transport organization, technical, human and collective risk factors. These risks should be evaluated by the physician before requesting a diagnostic or therapeutic procedure based on a risk-benefit analysis.^(^^1-3^^)^

Several studies have identified protective factors to minimize adverse events related to transport, such as equipment checks during transport, patient preparation, appropriate sedation and an experienced transport team. The incidence and severity of adverse events vary between studies. These discrepancies can be explained by differences in the definition of adverse events. The most clinically useful definition of an adverse event is an event that leads to a change in therapy during transport. It is worth noting that events can arise during transport or secondarily.^(^^4,5^^)^

The objectives of this study were to describe the incidence of clinical and non-clinical adverse events (e.g., infusion pump or communication failures) during the IHT of critically ill patients and to analyze the risk factors associated with these complications.

## METHODS

A cohort study was conducted with retrospective data collected from October 2016 to October 2017 in large hospital with 697 beds, including 6 adult ICUs (neurological, cardiac and general - 204 beds). All IHTs for diagnostic and therapeutic purposes of patients who were hospitalized in ICUs and needed transport to a diagnostic unit or the hemodynamic unit were analyzed. All transports were performed by a dedicated transport team consisting of an intensivist, a nurse, a nursing technician and, in the cases of patients under mechanical ventilation, a physical therapist. The study was approved by the Ethics Committee of the *Hospital Beneficência Portuguesa* (Nº 5966) and the informed consent term was waived.

The records of the consultations carried out in the study period were evaluated (Appendix 1), which were filled out by the doctor who performed the transport, and the adverse events occurring during transport and the clinical conditions were analyzed.

The clinical complications described were hemodynamic instability (systolic blood pressure < 90mmHg), respiratory instability (acute decrease in saturation < 90% and/or increase in respiratory rate > 24rpm), agitation, convulsive crisis or decreased consciousness level.

Non-clinical adverse events were related to communication and equipment problems (e.g., lack of continuity of care due to ineffective communication; delayed examination; and problems related to equipment batteries, infusion pumps and oxygen supply). The communication failures were mainly due to problems during the transfer of the clinical case among the multiprofessional team or failures between the ICU team and the diagnostic team, which caused delays in performing exams.

The data are presented as the means and standard deviations for the quantitative variables and as frequencies for the qualitative variables. The chi-square test (or Fisher's exact test) for the qualitative variables and the Mann-Whitney test for the quantitative variables were used to assess relationships between patient characteristics and the occurrence of at least one complication. Subsequently, to assess the relationships between the set of patient characteristics and the occurrence of complications, multiple logistic regression was used only for selected variables with a p value < 0.2 in the previous tests. In all studies, a p value of 0.05 was considered significant, and data were analyzed using the Statistical Package for Social Sciences (SPSS) v25.

## RESULTS

In the analyzed period, 1,559 transports of critically ill patients were performed with 1,348 patients. The mean age of the patients was 66 ± 17 years, with the male gender predominating (54.7%). The mean transport time was 43 ± 34 minutes. Of the sample, 19.8% were using vasoactive drugs during transport, and of these, 42.7% experienced an adverse event related to the clinical condition and 26.4% experienced a non-clinical adverse event. Sedatives were used in 13.7% of the patients, and 10.6% of the patients were under mechanical ventilation. The general characteristics of the patients are summarized in table 1.

**Table 1:** Characteristics of patients undergoing intrahospital transport

Variables	N (%)
Sex	
Female	706 (45.3)
Male	853 (54.7)
Type of hospitalization	
Surgical	609 (39.1)
Clinical	950 (60.9)
Invasive mechanical ventilation	166 (10.6)
Non-invasive mechanical ventilation	1 (0.1)
Use of sedatives	213 (13.7)
Dexmedetomidine	107 (6.9)
Fentanyl	38 (2.4)
Remifentanil	90 (5.8)
Propofol	49 (3.1)
Midazolam	64 (4.1)
Others	3 (0.2)
Use of vasoactive drugs	308 (19.8)
Noradrenaline	189 (12.1)
Dopamine	9 (0.6)
Dobutamine	82 (5.3)
Nitroprusside	40 (2.6)
Nitroglycerin	29 (1.9)
Vasopressin	4 (0.3)
Clinical condition at the end of transport	
Unchanged	1.530 (98.1)
Worse	29 (1.9)

Adverse events related to clinical situations occurred in 117 transports (7.5%), and there was more than one complication in 14 patients. Non-clinical events occurred in 125 transports (8.0%), and there was more than one complication in 13 patients (Table 2). Psychomotor agitation and hemodynamic instability were the most frequent clinical events. Among the non-clinical events, communication failures occurred in 99 transports.

**Table 2:** Clinical and non-clinical complications related to transport

Variables	N (%)
Clinical complications	
Hemodynamic instability	43 (2.8)
Respiratory insufficiency	21 (1.3)
Convulsive crisis	5 (0.3)
Psychomotor agitation	48 (3.1)
Decreased consciousness level	4 (0.3)
Other complications	10 (0.6)
Non-clinical complications	
Communication failure	99 (6.4)
Lack of oxygen	2 (0.1)
Infusion pump failure	4 (0.3)
Multi-parameter monitor battery failures	8 (0.5)
Delay in performing exams	25 (1.6)

Of the clinical events, 58.1% occurred in male patients, and 52.1% were aged 57 to 78 years. Using multivariate analysis (logistic regression) to identify the independent variables associated with complications, the use of sedatives, noradrenaline and nitroprusside and a transport time over 36.5 minutes were related to clinical complications. The use of dobutamine and a transport time greater than 36.5 minutes were related to non-clinical events (Tables 3 and 4).

**Table 3:** Logistic regression related to clinical complications during intrahospital transport

	Odds ratio (95%CI)	p value
Dexmedetomidine	2.72 (1.51 - 5.08)	0.001
Fentanyl	3.07 (1.30 - 7.24)	0.01
Remifentanil	2.17 (1.10 - 4.28)	0.02
Propofol	2.68 (1.23 - 5.82)	0.01
Noradrenaline	2.09 (1.25 - 3.51)	0.005
Nitroprusside	3.98 (1.71 - 9.25)	0.001
Transport time > 36.5 minutes	1.67 (1.11 - 2.52)	0.01

95%CI - 95% confidence interval.

**Table 4:** Logistic regression related to non-clinical complications during intrahospital transport

	Multivariate analysis Odds ratio (95%CI)	p value
Dobutamine	2.24 (1.19 - 4.20)	0.001
Transport time > 36.5 minutes	1.67 (1.11 - 2.52)	0.01

95%CI - 95% confidence interval.

In the transports associated with non-clinical events, 55.2% of the patients were male, and 66.4% had a transport time greater than 36.5 minutes. Additionally, 16.8% of the patients were under mechanical ventilation, 20.8% used sedatives and 26.4% used vasoactive drugs. Failure in communication among health professionals involved in the transport of critically ill patients, mainly related to information exchange, was the most frequent problem, occurring in 6.4% of the transports.

At the end of transport, 98.1% of the patients presented clinical conditions unchanged relative to baseline. Clinical events during transport led to an increase in the length of stay in the ICU and hospital in only one patient (0.1%). No patient had an increased mechanical ventilation time, and no deaths were related to transport complications (Table 1S - Supplementary material).

## DISCUSSION

Intrahospital transport is related to a high incidence of complications and adverse events, with a negative impact on clinical outcomes.^(^^1-3^^)^

In this sample, we observed a low rate of adverse events, both clinical and non-clinical, at levels lower than those found in most studies, with complications observed in up to 79% of transported patients.^(^^3-6^^)^

A recent study^(^^4^^)^ demonstrated that the clinical condition pre-transport is an independent risk factor for the occurrence of complications during transport. In our study, gender, age and type of hospitalization (clinical or surgical) were not related to complications, but the use of mechanical ventilation and the use of vasoactive drugs and sedatives were related to adverse events.

The non-clinical events most commonly described were related to communication, which can reach rates of up to 60% during IHT.^(^^3^^)^ In our series, communication was the main non-clinical event observed, with 99 occurrences. However, in the logistic regression analysis, transport time and use of dobutamine were related to non-clinical events.

It is important to ensure that transport is performed by professionals trained in the task, which guarantees a lower rate of complications and better outcomes.^(^^5,7-9^^)^ In our study, the team responsible for IHT was dedicated exclusively to this function and was composed of a multiprofessional team working in intensive care.

The most commonly reported team-related adverse events are communication failures, which can reach 60%.^(^^8^^)^ In the present study, the rate of non-clinical complications was 7.2%, a number significantly lower than that found in the international literature. The presence of a team trained in monitoring during the entire transport is associated with the safety of the patient being transported.^(^^5,7-9^^)^

There is disagreement in the literature regarding the occurrence of adverse events and the duration of transport.^(^^10-12^^)^ However, in our study, we demonstrated an association between transport time and clinical complications, with transport times greater than 36.5 minutes being related to a higher incidence of complications.

Adverse events are related to worse outcomes during a hospital stay.^(^^13-17^^)^ Better processes and professional training can reduce the occurrence of these events and contribute to shorter hospital stays and lower hospitalization costs.^(^^16^^)^ Worse outcomes related to complications during transport were not observed in our sample.

The limitations of the study were the following: being a single-center study; the possible failures in filling out the medical records, which may have caused underreporting of adverse events; and the low number of complications and adverse events, which limits the accuracy of risk factor identification.

## CONCLUSION

Intrahospital transport is related to a high incidence of adverse events. Transport time and use of sedatives and vasoactive drugs were associated with such events.

## Appendix 1 Transport group service sheet

**Table:**

			Label
**MEDICAL RECORD**			

**Transport Group**			

Date of service: ____/____/______			
Call time: _____:____h Start time: ____:____h			

Reason for hospitalization: __________________________________________________________________________________________________________			
Exam requested: _________________________________________________________________________________________________________________			
( ) with sedation ( ) without sedation			
Reason for exam: ________________________________________________________________________________________________________________			

**EXIT FROM THE ICU:**			
Time: ________			
BP: _________mmHg HR: _____BPM RR: _______BPM SatO_2_: ________________			
Intensivist responsible for leaving the ICU: _______________________________________________________________________________________________			

**Vasoactive drugs:**			

**Drug**		**Dose (mcg/kg/min)**	
Noradrenaline			
Dopamine			
Dobutamine			
Vasopressin			
Others:			

**Sedation:**			

**Drug**		**Dose (mcg/kg/min)**	
Propofol			
Midazolam			
Fentanyl			
Remifentanil			
Dexmedetomidine			
Others:			

Complications during **transport**: ( ) no ( ) yes			
If yes, please describe:			
________________________________________________________________________________________________________________________________________________________________________________			

Complications during the **exam/procedure**: ( ) no ( ) yes			
If yes, please describe:			
________________________________________________________________________________________________________________________________________________________________________________			

**RETURN TO ICU:**			
Time: ________			
BP: _________mmHg HR: _____BPM RR: _______BPM SatO_2_: ______________			
Intensivist responsible for return to ICU: ___________________________________			

Was there a need to change the dosage of vasoactive drugs? If so, which one(s)?			
________________________________________________________________________________________________________________________________________________________________________________			

Was there a need to change the dosage of sedatives? If so, which one(s)?			
________________________________________________________________________________________________________________________________________________________________________________			

Physician responsible for transportation: ________________________________________________________________________________________________			
