# Supplementary material for: Adverse events during intrahospital transport of critically ill patients in a large hospital
Source: Rev Bras Ter Intensiva. 2019 Jan-Mar;31(1):15–20. doi: 10.5935/0103-507X.20190003 (PMC6443312; doi:10.5935/0103-507X.20190003)
Supplement: Supplementary file 1 [file rbti-31-01-0015-suppl1.pdf]

Adverse events during intrahospital transport of critically ill patients in a large hospital

Eventos adversos durante transporte intra-hospitalar de pacientes críticos em hospital de grande porte

Viviane Cordeiro Veiga[1,2], Natalia Fioravanti Postalli[2], Thais Kawagoe Alvarisa[2], Phillipe Pereira Travassos[2], Raquel Telles da Silva Vale[2], Cleyton Zanardo de Oliveira[3], Salomón Soriano Ordinola Rojas[1,2]

Table 1S - Relationship between patient characteristics and the occurrence of clinical and non-clinical complications (univariate analysis)

	Clinical complications			Non-clinical complications		
	No<br>N (%)	Yes<br>N (%)	p value	No<br>N (%)	Yes<br>N (%)	p value
Sex						
Female	657 (45.6)	49 (41.9)	0.442	650 (45.3)	56 (44.8)	0.909
Male	785 (54.4)	68 (58.1)		784 (54.7)	69 (55.2)	
Age						
≤ 57	391 (27.1)	31 (26.5)	0.285	391 (27.3)	31 (24.8)	0.836
> 57 and <78	656 (45.5)	61 (52.1)		658 (45.9)	59 (47.2)	
≥ 78	395 (27.4)	25 (21.4)		385 (26.8)	35 (28.0)	
Type of hospitalization						
Surgical	560 (38.8)	49 (41.9)	0.516	564 (39.3)	45 (36.0)	0.464
Clinical	882 (61.2)	68 (58.1)		870 (60.7)	80 (64.0)	
Mechanical ventilation						
No	1.314 (91.1)	79 (67.5)	< 0.001	1.289 (89.9)	104 (83.2)	0.020
Yes	128 (8.9)	38 (32.5)		145 (10.1)	21 (16.8)	
Non-invasive ventilation						
No	1,441 (99.9)	117 (100.0)	0.999	1.433 (99.9)	125 (100.0)	0.999
Yes	1 (0.1)	0 (0.0)		1 (0.1)	0 (0.0)	
Sedation						
No	1,279 (88.7)	67 (57.3)	< 0.001	1.247 (87.0)	99 (79.2)	0.015
Yes	163 (11.3)	50 (42.7)		187 (13.0)	26 (20.8)	
Dexmedetomidine						
No	1,361 (94.4)	91 (77.8)	< 0.001	1.340 (93.4)	112 (89.6)	0.103
Yes	81 (5.6)	26 (22.2)		94 (6.6)	13 (10.4)	
Fentanyl						
No	1.413 (98.0)	108 (92.3)	0.001	1.403 (97.8)	118 (94.4)	0.028
Yes	29 (2.0)	9 (7.7)		31 (2.2)	7 (5.6)	
Remifentanil						
No	1.379 (95.6)	90 (76.9)	< 0.001	1.355 (94.5)	114 (91.2)	0.130
Yes	63 (4.4)	27 (23.1)		79 (5.5)	11 (8.8)	
Propofol						
No	1408 (97.6)	102 (87.2)	< 0.001	1.392 (97.1)	118 (94.4)	0.107
Yes	34 (2.4)	15 (12.8)		42 (2.9)	7 (5.6)	

Continue...

... continuation

	Clinical complications			Non-clinical complications		
	**No**	**Yes**	**p value**	**No**	**Yes**	**p value**
	**N (%)**	**N (%)**		**N (%)**	**N (%)**	
Midazolam						
No	1.391 (96.5)	104 (88.9)	0.001	1.379 (96.2)	116 (92.8)	0.069
Yes	51 (3.5)	13 (11.1)		55 3.8)	9 (7.2)	
Vasoactive drugs						
No	1.185 (82.2)	66 (56.4)	< 0.001	1.159 (80.8)	92 (73.6)	0.052
Yes	257 (17.8)	51 (43.6)		275 (19.2)	33 (26.4)	
Noradrenaline						
No	1.292 (89.6)	78 (66.7)	< 0.001	1.266 (88.3)	104 (83.2)	0.095
Yes	150 (10.4)	39 (33.3)		168 (11.7)	21 (16.8)	
Dopamine						
No	1.434 (99.4)	116 (99.1)	0.505	1.427 (99.5)	123 (98.4)	0.159
Yes	8 (0.6)	1 (0.9)		7 (0.5)	2 (1.6)	
Dobutamine						
No	1.375 (95.4)	102 (87.2)	< 0.001	1.365 (95.2)	112 (89.6)	0.007
Yes	67 (4.6)	15 (12.8)		69 (4.8)	13 (10.4)	
Sodium nitroprusside						
No	1.410 (97.8)	109 (93.2)	0.008	1.395 (97.3)	124 (99.2)	0.367
Yes	32 (2.2)	8 (6.8)		39 (2.7)	1 (0.8)	
Nitroglycerin						
No	1.414 (98.1)	116 (99.1)	0.719	1.406 (98.0)	124 (99.2)	0.725
Yes	28 (1.9)	1 (0.9)		28 (2.0)	1 (0.8)	
Transport Time						
≤ 36.5 minutes	747 (51.8)	43 (36.8)	0.002	748 (52.2)	42 (33.6)	< 0.001
> 36.5 minutes	695 (48.2)	74 (63.2)		686 (47.8)	83 (66.4)	
